# HRCT score in bronchiectasis: Correlation with pulmonary function tests and pulmonary artery pressure

**DOI:** 10.4103/1817-1737.39675

**Published:** 2008

**Authors:** Abdullaziz H. Alzeer

**Affiliations:** *Department of Medicine, King Khalid University Hospital, Riyadh, Saudi Arabia*

**Keywords:** Bronchiectasis, high-resolution CT, pulmonary artery pressure

## Abstract

**BACKGROUND:**

High-resolution CT scan (HRCT) and its score have an important role in delineating pathological changes and pulmonary functional impairment in patients with bronchiectasis.

**AIMS:**

To assess pulmonary function tests (PFTs) in patients with cystic and cylindrical bronchiectasis. To correlate HRCT score with PFTs and systolic pulmonary artery pressure (SPAP) in both radiological types.

**MATERIALS AND METHODS:**

A cross-sectional study of patients with bronchiectasis diagnosed by HRCT was conducted at King Khalid University Hospital, Riyadh, Saudi Arabia. PFTs, HRCT score and SPAP were measured in both types.

**RESULTS:**

We studied 94 patients with bronchiectasis; 62 were cystic and 32 were cylindrical. Their mean age was 53.4 ± 17.5 SD years. Forced vital capacity (FVC%) and forced expiratory volume in 1 second (FEV1%) were significantly lower in cystic patients (*P* < 0.0001) as compared with cylindrical patients; and diffusion capacity of carbon monoxide (DLCO%) was also significantly lower (*P* < 0.01). In the cystic group, PaO_2_ was significantly lower; and PaCO_2_, higher (*P* < 0.0001). HRCT score was correlated with FEV1% (r = −0.51). HRCT score was significantly lower in the cystic group (*P* = 0.002) and correlated with SPAP (*r* = 0.23). Global HTCT score of 10.3 ± 2.5 was associated with SPAP ≥40 mm Hg (*P* = 0.011).

**CONCLUSION:**

Patients with cystic bronchiectasis have significantly higher impairment of pulmonary physiology as compared with those with cylindrical bronchiectasis patients. HRCT score correlated with PFTs and SPAP.

Bronchiectasis is a clinical syndrome characterized by repetitive lung infection and manifested by cough and excessive sputum production.[1,2] Due to disturbed pulmonary physiology, these patients may show airflow obstruction or mixed obstructive and restrictive defect.[3–5] High-resolution CT scan of the lung (HRCT) is proven to be a highly sensitive noninvasive technique for delineating the bronchiectatic segments.[6,7]

A previous study demonstrated the correlation between HRCT score and forced vital capacity in 1 second (FEV_1_),[8] while Lynch *et al.* correlated it with the extent of physiological impairment.[9] However, differentiation between patients with cystic and cylindrical bronchiectasis using HRCT score and these physiological parameters were sparsely discussed.[9] Furthermore, there are no data on HRCT score and SPAP. We therefore carried out a cross-sectional study to (1) assess the degree of pulmonary function test (PFT) impairment in both cystic and cylindrical bronchiectasis, (2) correlate these parameters to HRCT score, and (3) explore the relationship between SPAP and HRCT score in both the groups of patients.

## Materials and Methods

### Patients

The study sample comprised 94 stable patients with bronchiectasis diagnosed by HRCT and recruited from the outpatient clinic at King Khalid University Hospital, Riyadh, Saudi Arabia. Consecutive patients with diagnosis of bronchiectasis were included. The study was approved by the local research committee, and consent was taken from each patient. Exclusion criteria were (1) age less than 14 years, (2) smoker or ex-smoker, (3) mixed disease on HRCT, (4) had previous resectional lung surgery, and (5) ischemic heart disease, valvular heart disease, or hypertension.

Data collected included age, sex, and respiratory symptoms such as cough, sputum, hemoptysis, and shortness of breath or wheezes. All patients were tested for α-1 anti-trypsin and immunoglobulin levels, and sputum was tested for acid-fast bacilli (stain and culture).

### Pulmonary function test

Measurement of forced vital capacity (FVC) and forced vital capacity in 1 second (FEV_1_; best results of 3 successful attempts) was done in all patients. Total lung volume (TLC) and residual volume (RV) were measured by plethysmography. Diffusion capacity of carbon monoxide (DLCO) was measured by single-breath technique (671178, Erich Jaeger Master screen PFT, GmbH D-9204 Hoechben, Germany). Data were expressed as percentage of predicted value using the standard protocol of the American Thoracic Society.[10] Arterial blood gases were measured while patients were breathing room air. The alveolar-arterial oxygen gradient [p (A-a) O_2_] was calculated from the alveolar gas equation assuming a standard respiratory exchange ratio of 0.8. Hypoxemia and hypercarpia were defined as PaO_2_ < 60 mm Hg and PaCO_2_ >45 mm Hg respectively.

### Echocardiography

Two-dimensional transthoracic cardiographs with color flow imaging were performed in all patients (Philips model 5500). The ECHO study was read by a cardiologist without information about patient status. SPAP was calculated based on the modified Bernoulli equation, and right atrial pressure was estimated as 5, 10, 15, or 20 mm Hg on the basis of size and respiratory changes of the inferior vena cava using previously described techniques.[11] Pulmonary hypertension (PH) was defined in this study as SPAP ≥40 mm Hg based on criteria established by the World Health Organization Symposium on Primary Pulmonary Hypertension (1998).

### HRCT

All patients underwent HRCT of the chest (GE Medical Systems, Light Speed L52002). High-resolution images were obtained in full inspiration for all patients, while expiratory images were acquired for patients suspected to have small airway disease. High-resolution images were obtained at 1 mm collimation at 1-mm intervals from the apices to the lung bases. Images were reconstructed into a bow algorithm using standard window setting (window level: 700 HU; window width: 1500 HU). The CT scan was interpreted for the presence of bronchiectasis severity, pattern, distribution, and associated disease processes such as emphysema and small airway disease by 2 radiologists who were blinded to other clinical and laboratory results; decisions were reached by consensus. The presence of bronchiectasis was based on (1) lack of tapering of bronchi, (2) bronchial dilatation when its internal diameter was at least 110% larger than the adjacent pulmonary artery, or (3) visualization of the peripheral bronchi within 1 cm of the costal pleural surface or adjacent mediastinal pleural surface.[12] Cylindrical bronchiectasis was diagnosed based on dilatation and thickening of the bronchial wall :arterial wall ratio >1[13]; and cystic bronchiectasis was diagnosed by noticing thin-walled cystic spaces that may contain fluid and these were seen in subsequent axial cuts either in a conglomerate fashion or in branching order.[14] Six CT scan criteria were assessed: (1) bronchial dilatation, (2) peribronchial wall thickening, (3) number of bronchiectasis segments, (4) number of bullae, (5) number of emphysema segments, and (6) criteria of associated small airway disease. Each of the above parameters were scored from 0 in absence of a lesion through 3 according to severity of disease, with the small airway disease scored as 0 if it was absent or 1 if it was present. A global score for each patient was calculated from these measures that reflected the radiological overall severity of the disease process and its associations, with a maximum possible score of 16.[15] A bulla was defined as a sharp demarcated area of emphysema measuring ≥1 cm in diameter and possessing a wall <1 mm in thickness. Emphysema was defined as (1) an area of low attenuation in comparison with continuous normal lung parenchyma, with vascular disruption and lacking a well-defined wall; or (2) an area of low attenuation possessing a wall less than 1 mm in thickness. Criteria for associated small airway abnormalities included small centrilobular opacities, tree-in-bud opacities, bronchiectasis, air trapping, and mosaic perfusion.[16]

### Statistical analysis

Data were analyzed using SPSS statistical software for windows (version 10.0). Continuous variables that followed a normal distribution were summarized as mean ± SD. Student *t* test (two-tailed) was used to compare the means of 2 groups, whereas chi-square test was used to evaluate associations between categorical variables. Pearson's correlation coefficient was calculated to quantify the relationship of 2 continuous variables, and simple linear regression analysis was done to quantify the linear relationship between a continuous outcome and an independent variable.

## Results

The total number of patients was 94, of whom 29 (30.8%) were men. The mean (SD) age was 53.4 (17.7) years. Cystic bronchiectasis was diagnosed in 62 (66%) and cylindrical in 32 (34%) patients. Pulmonary function test data are shown in Table 1. There was a statistically significant difference between cystic and cylindrical groups in relation to the mean values of FVC%, FEV1% (*P* < 0.0001), FEV1/FVC% (*P value* < 0.03), DLCO% (*P* < 0.009). In cystic group, PaO_2_ was significantly lower; and PaCO_2_, higher (*P* < 0.0001). The calculated A-a gradient was high. There was also a statistically significant difference in mean global HRCT scores for cystic compared with cylindrical patients (*P* < 0.002). No statistically significant differences were found between groups for mean TLC percentage of predicted or residual volume percentage of predicted. Five patients with cylindrical bronchiectasis had normal spirometry, and 21 (22.3%) patients used home oxygen; of these, 20 patients had cystic bronchiectasis.

**Table 1:** Comparison of results of pulmonary function test between cystic and cylindrical patients*

Variables	All (*n* = 94)	Cystic (*n* = 62)	Cylindrical (*n* = 32)	*P*-value
VC%	65.8 ± 21.4	58.8 ± 19.6	79.0 ± 18.4	<0.0001†
FEVI%	59.7 ± 23.2	53.4 ± 21.6	71.6 ± 21.7	<0.0001†
FEVI/FVC	74.5 ± 6.6	73.5 ± 7.3	76.4 ± 4.9	0.03†
TLC%	91.1 ± 22.4	87.9 ± 18.6	97.3 ± 27.8	0.11
RV%	134.5 ± 59.5	131.1 ± 51.9	140.3 ± 71.5	0.57
DLCO%	66.7 ± 25.9	61.1 ± 25.5	76.9 ± 24.0	0.009†
A-a gradient	24.8 ± 6.6	24.9 ± 7.3	24.6 ± 5.2	0.88
PCO_2_	44.2 ± 11.3	47.6 ± 12.2	37.9 ± 4.9	<0.0001†
PO_2_	66.2 ± 14.1	62.3 ± 13.6	73.8 ± 11.8	<0.0001†
Global Score	9.1 ± 2.8	9.8 ± 2.7	7.9 ± 2.7	0.002†

* Data are in mean ± S.D

† Statistically significant

**Figure 1:**
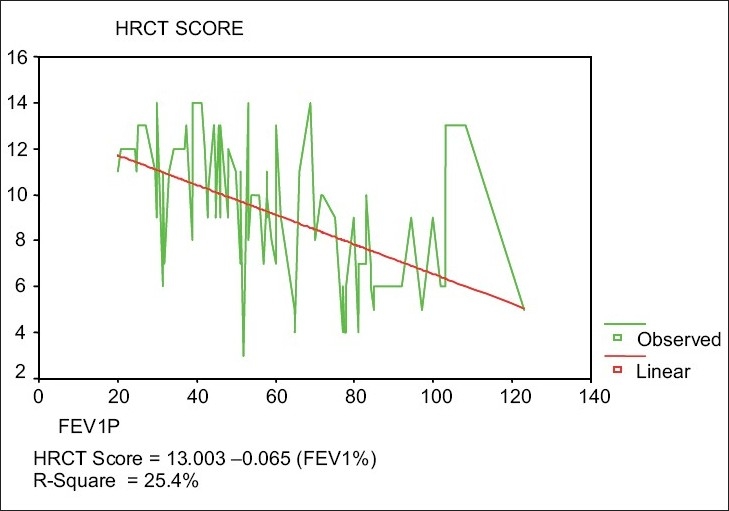
Relationship between HRCT Global Score and FEV1% of all patients

Global HRCT scores for all cystic and cylindrical patients were statistically significantly negatively correlated with the values of FEV1% (*r* = –0.51; *P* < 0.05). There was a statistically significant negative linear relationship between global HRCT scores of all patients [Figure 1], where 25.4% of variability in global HRCT values was explained by the change of FEV1 values. Similar negative linear relationships were observed for both cystic and cylindrical group patients.

**Figure 2:**
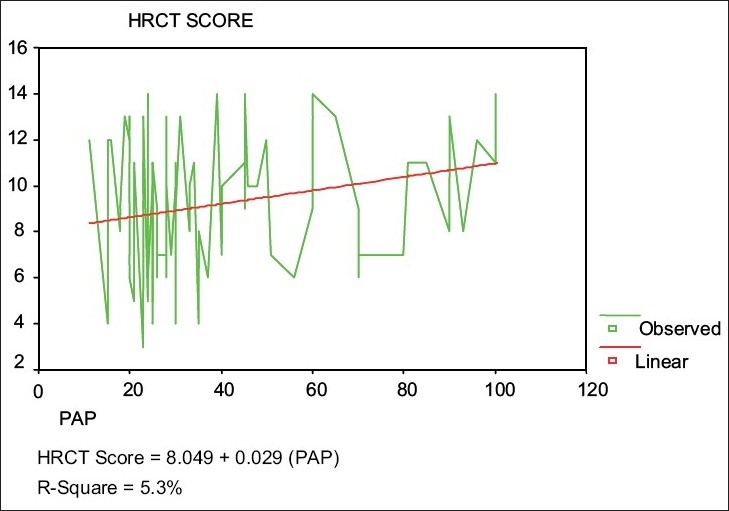
Relationship between HRCT Global Score and PAP of all patients

SPAP was significantly positively correlated with the global score of all patients (*r* = 0.23). There was a statistically significant positive linear relationship between global scores and SPAP values for all patients (*P* < 0.05) [Figure 2]. The mean global score (8.67 ± 2.9) for patients whose SPAP value was < 40 mm Hg was significantly less than that (10.3 ± 2.5) of patients whose SPAP value was ≥40 mm Hg *(P* < 0.011).

Of 31 patients who had ≥5 lobes, 26 (83.9%) had cystic bronchiectasis, whereas 5 (16.1%) had cylindrical bronchiectasis (*P* < 0.019) [Table 2].

**Table 2:** Association between number of lobes and type of bronchiectasis*

No. of lobes	Cystic *n* (%)	Cylindrical *n* (%)
≥5	26 (83.9)	5 (16.1)
<5	36 (57.1)	27 (42.9)

* X^2^ = 5.47; *P* < 0.019

## Discussion

This study compared PFT results with HRCT score in patients with bronchiectasis. Patients with cystic bronchiectasis had significantly worse PFTs and higher global HRCT scores than patients with cylindrical bronchiectasis. The global HRCT score was significantly correlated with SPAP.

Obstructive pulmonary defect is reported in a majority of patients with bronchiectasis, and a mixed obstructive/restrictive defect is uncommon.[3,4] In this study, FVC% and FEV_1_% were significantly higher in patients with cylindrical bronchiectasis as compared with the cystic group [Table 1].

Lynch *et al.* showed that airflow obstruction was common in 33 patients with pure cylindrical disease; however, the flows of their patients were lower than those in the patients in the current study and were partially attributed to smoking.[9] Our study is in agreement with their report; however, our patients were nonsmokers, suggesting that other underlying pathology was the cause of airflow obstruction, most likely bronchiolitis, secretion, and bronchial hyper-responsiveness.[17,18] Hansel *et al.* studied areas of decreased attenuation using HRCT and concluded that small airway disease and inflammatory bronchiolitis are integral part of bronchiectasis. In their study, bronchiolitis was also found in lobes that were free of bronchiectasis, suggesting that bronchiolitis may precede the development of bronchiectasis[17] and that small airways are affected before large airways.[19]

The cystic group had significantly lower DLCO% and FVC% than the cylindrical group. These further support the restrictive component of their disease, which is presumably related to atelectasis, bullae, emphysema, and fibrosis, representing a late irreversible disease commonly seen in these patients.[16]

Air trapping is also an important component of bronchiectasis.[16,20] The association between bronchiectasis and air trapping was studied by Kang *et al.* using CT pathology. Their findings of hyperlucency on expiratory CT adjacent to bronchiectasis signify inflammatory bronchiolitis.[16] In this study, air trapping was present in nearly all patients. RV% was high but was not statistically significantly different between cystic and cylindrical groups [Table 1]. The presence of air trapping in bronchiectasis would support the notion that inflammatory bronchiolitis contributes to air flow obstruction.

Hypoxemia and hypercapnia were more significantly seen in cystic group [Table 1], and majority of them were on ‘home’ oxygen. The mechanism of hypoxemia in these patients was likely due to the combination of low ventilation perfusion (V/Q) mismatch and right-to-left shunt, since their A-a gradient was high. A previous study of regional ventilation in bronchiectatic patients using Xenon-133 scintigraphy demonstrated reduced ventilation that was widespread even in nonbronchiectasis areas.[21]

Ashore studied hemodynamics in bronchiectasis using V/Q scan and pulmonary angiography, and proposed 2 types of bronchiectasis: perfused (cylindrical) and nonperfused (cystic).[22] He found that the latter group had more capillary bed destruction and extensive anastomosis between pulmonary and bronchial arteries, with greater likelihood of compromised gas exchange.[22] Furthermore, in advanced bronchiectasis the respiratory muscles are exposed repetitively to increased work because of hyperinflation and alteration of the length-tension relationship.[23] A previous study demonstrated an expiratory flow limitation in patients with bronchiectasis, which further contributes to ventilatory defect in these patients.[8]

In this study, patients with cystic disease were found to have significantly higher HRCT scores, with more extensive disease and more bronchiectatic lobe involvement [Table 2]. We also found a correlation between HRCT score and FEV_1_%(r = −0.51) [Figure 1]. This is in agreement with previous reports; as the HRCT score reflects bronchiolitis, areas of emphysema, bullae, and bronchiectatic segments[8,9] - all can affect FEV_1_% measurements.[16,24]

Spirometry results were normal in 5 patients with cylindrical disease while CT scans depicted structural abnormalities - this observation was reported before - since CT scans directly quantify lung structural abnormalities associated with bronchiectasis and PFTs indirectly measure pulmonary function to give a global assessment and can be even normal in milder disease.[25]

PH is commonly seen in advanced bronchiectasis, particularly with cystic disease.[26] We reported recently that 32.9% of stable patients with bronchiectasis had PH and found that SPAP was higher in cystic bronchiectasis with concomitant right and left ventricular dysfunction.[26] In this study, SPAP was positively correlated with HRCT score (*r* = 0.23) [Figure 2]. The pathogenesis of PH in these patients is related to impairment of pulmonary physiology due to extensive lung damage and high pulmonary vascular resistance. This is presumably due to extensive anastomosis between pulmonary and bronchial arteries and the presence of hypoxemia, which further contribute to higher SPAP.[22,26] HRCT depicts these pathological changes. To the best of the author's knowledge, this is the first report in the medical literature on the correlation between HRCT score and SPAP. Moreover, high HRCT score not only reflects lung damage in these patients, but it can also be associated with PH.

An important limitation of this study is the fact that it is a cross-sectional study of stable patients with bronchiectasis. Future studies should address the correlation of CT scan with long-term outcome and its role in acute exacerbations.[27] There are also limitations to the use of echocardiography in evaluating SPAP in patients with advanced lung disease. Nevertheless, Doppler echocardiograph is a convenient noninvasive and relatively accurate tool for evaluating SPAP.

In conclusion, cystic bronchiectasis is associated with more severe lung function impairment and worse HRCT scores as compared with cylindrical bronchiectasis. HRCT scores correlate with FEV1% and SPAP and could be a predictor of future PH.

## References

[1] Reid LM (1950). Reduction in bronchial subdivision in bronchiectasis. Thorax.

[2] Kolbe J, Wells AU (1996). Bronchiectasis: A neglected cause of respiratory morbidity and mortality. Respirology.

[3] Pande JN, Jain BP, Gupta RG, Guleria JS (1971). Pulmonary ventilation and gas exchange in bronchiectasis. Thorax.

[4] Ip M, Lauder IJ, Wong WY, Lam WK, So SY (1993). Multivariate analysis of factors affecting pulmonary function in bronchiectasis. Respiration.

[5] Cherniack NS, Carton RW (1966). Factors associated with respiratory insufficiency in bronchiectasis. Amer J Med.

[6] Grenier P, Maurice F, Musset D, Menu Y, Nahum H (1986). Bronchiectasis: Assessment by thin-section CT. Radiology.

[7] Silverman PM, Godwin JD (1987). CT-bronchographic correlations in bronchiectasis. J Comput Assist Tomogr.

[8] Koulouris NG, Retsou S, Kosmas E, Dimakou K, Malagari K, Mantzikopoulos G (2003). Tidal expiratory flow limitation, dyspnoea and exercise capacity in patients with bilateral bronchiectasis. Eur Respir J.

[9] Lynch DA, Newell J, Hale V, Dyer D, Corkery K, Fox NL (1999). Correlation of CT findings with clinical evaluations in 261 patients with symptomatic bronchiectasis. AJR Am J Roentgenol.

[10] American Thoracic Society (1995). Standardization of Spirometry, 1994 Update. Am J Respir Crit Care Med.

[11] Currie PJ, Seward JB, Chan KL, Fyfe DA, Hagler DJ, Mair DD (1985). Continuous wave Doppler determination of right ventricular pressure: A simultaneous Doppler-catheterization study in 127 patients. J Am Coll Cardiol.

[12] Smith IE, Jurriaans E, Diederich S, Ali N, Shneerson JM, Flower CD (1996). Chronic sputum production: Correlations between clinical features and findings on high resolution computed tomographic scanning of the chest. Thorax.

[13] Kim JS, Müller NL, Park CS, Grenier P, Herold CJ (1997). Cylindrical bronchiectasis: Diagnostic findings on thin-section CT. AJR Am J Roentgenol.

[14] Marti-Bonmati L, Catala FJ, Ruiz Perales F (1991). Computed tomography differentiation between cystic bronchiectasis and bullae. J Thorac Imaging.

[15] Roberts HR, Wells AU, Milne DG, Rubens MB, Kolbe J, Cole PJ (2000). Airflow obstruction in bronchiectasis: Correlation between computed tomography features and pulmonary function tests. Thorax.

[16] Kang EY, Miller RR, Müller NL (1995). Bronchiectasis: Comparison of preoperative thin-section CT and pathologic findings in resected specimens. Radiology.

[17] Hansell DM, Wells AU, Rubens MB, Cole PJ (1994). Bronchiectasis: Functional significance of areas of decreased attenuation at expiratory CT. Radiology.

[18] Bahous J, Cartier A, Pineau L, Bernard C, Ghezzo H, Martin RR (1984). Pulmonary function tests and airway responsiveness to methacholine in chronic bronchiectasis of the adult. Bull Eur Physiopathol Respir.

[19] Bedrossian CW, Greenberg SD, Singer DB, Hansen JJ, Rosenberg HS (1976). The lung in cystic fibrosis: A quantitative study including prevalence of pathologic findings among different age groups. Hum Pathol.

[20] Robinson TE, Leung AN, Northway WH, Blankenberg FG, Bloch DA, Oehlert JW (2001). Spirometer-triggered high-resolution computed tomography and pulmonary function measurements during an acute exacerbation in patients with cystic fibrosis. J Pediatr.

[21] Bass H, Henderson JA, Heckscher T, Oriol A, Anthonisen NR (1968). Regional structure and function in bronchiectasis: A correlative study using bronchography and 133Xe. Am Rev Respir Dis.

[22] Ashour M (1996). Hemodynamic alterations in bronchiectasis: A base for a new subclassification of the disease. J Thorac Cardiovasc Surg.

[23] Braun NM, Arora NS, Rochester DF (1982). Force-length relationship of the normal human diaphragm. J App Physiol.

[24] Loubeyre P, Paret M, Revel D, Wiesendanger T, Brune J (1996). Thin-section CT detection of emphysema associated with bronchiectasis and correlation with pulmonary function tests. Chest.

[25] Helbich TH, Heinz-Peer G, Fleischmann D, Wojnarowski C, Wunderbaldinger P, Huber S (1999). Evolution of CT findings in patients with cystic fibrosis. AJR Am J Roentgenol.

[26] Alzeer AH, Al-Mobeirek AF, Al-Otair HA, Elzamzamy UA, Joherjy IA, Shaffi AS (2008). Right and left ventricular function and pulmonary artery pressure in patients with bronchiectasis. Chest.

[27] Arcasoy SM, Christie JD, Ferrari VA, Sutton MS, Zisman DA, Blumenthal NP (2003). Echocardiographic assessment of pulmonary hypertension in patients with advanced lung disease. Am J Respir Crit Care Med.

